# Production of adaptive movement patterns *via* an insect inspired spiking neural network central pattern generator

**DOI:** 10.3389/fncom.2022.948973

**Published:** 2022-11-18

**Authors:** Fabian Steinbeck, Thomas Nowotny, Andy Philippides, Paul Graham

**Affiliations:** ^1^Insect Navigation Group, School of Life Sciences, University of Sussex, Brighton, United Kingdom; ^2^AI Research Group, School of Engineering and Informatics, University of Sussex, Brighton, United Kingdom

**Keywords:** insect navigation, spiking neural network, central pattern generator, small scale search behavior, movement control, adaptive control, Lateral Accessory Lobe

## Abstract

Navigation in ever-changing environments requires effective motor behaviors. Many insects have developed adaptive movement patterns which increase their success in achieving navigational goals. A conserved brain area in the insect brain, the Lateral Accessory Lobe, is involved in generating small scale search movements which increase the efficacy of sensory sampling. When the reliability of an essential navigational stimulus is low, searching movements are initiated whereas if the stimulus reliability is high, a targeted steering response is elicited. Thus, the network mediates an adaptive switching between motor patterns. We developed Spiking Neural Network models to explore how an insect inspired architecture could generate adaptive movements in relation to changing sensory inputs. The models are able to generate a variety of adaptive movement patterns, the majority of which are of the zig-zagging kind, as seen in a variety of insects. Furthermore, these networks are robust to noise. Because a large spread of network parameters lead to the correct movement dynamics, we conclude that the investigated network architecture is inherently well-suited to generating adaptive movement patterns.

## 1. Introduction

A key component of the adaptive behavior of natural and artificial systems is motor control. Adaptive behavior often requires agents to produce active movement strategies to both acquire useful sensory information and then use it to their advantage. For instance, in visually guided behaviors in insects, we see many examples of movement strategies that can be described as active vision: saccadic flight structure in bees that helps extracting visual depth information (Wagner, 1986), peering in locusts (Wallace, 1959), and whole body rotational scanning movements in navigating ants (Wystrach et al., 2014). Some of these behaviors are examples of a prevalent type of search whereby individuals are seeking behaviorally relevant sensory information, e.g., zig-zagging in visually guided landing in bees and wasps (Lehrer and Collett, 1994; Collett et al., 2016) and sinuous trajectories during visual homing in ants (Buehlmann et al., 2018). Such active behaviors can be shown to increase performance in simulated or robot navigation (Kodzhabashev and Mangan, 2015; Steinbeck et al., 2020a,b) and are also seen in insects “searching” for behaviorally relevant information from other modalities such as moths searching for pheromones (Kanzaki and Mishima, 1996; Mishima and Kanzaki, 1999; Pansopha et al., 2014).

One particular area in the insect brain, the Lateral Accessory Lobe (LAL), has been shown to be a key component in the generation of search related motor signals (Kanzaki et al., 1992). It is commonly referred to as a pre-motor area and is the gateway for signals from higher brain areas on their way to motor centers (Namiki et al., 2014; Namiki and Kanzaki, 2016). It has been hypothesized that the LAL transforms navigation-related sensory signals into turning signals, which then can be realized by the motor systems downstream (Bidwell and Goodman, 1993; Zorović and Hedwig, 2013; Bidaye et al., 2020; Rayshubskiy et al., 2020). However, it also appears to generate intrinsic motor patterns in the absence of strong sensory input.

If the incoming sensory signals to the LAL are strong and reliable, these are directly passed on to the motor system for targeted steering. This has been demonstrated in crickets, where the LAL is involved in initiating the movements to steer toward the calls of conspecifics (phonotaxis). The steering seems to be initiated by a difference of firing rate between the left and right descending neurons (Zorović and Hedwig, 2013; Rayshubskiy et al., 2020). However, if the incoming signals are weak and unreliable, the LAL appears to be capable of intrinsically generating a rhythmic signal, which results in alternating turning directions as seen in silkmoths, which initiate a zig-wag walking behavior when losing a pheromone plume (Kanzaki et al., 1992). Here, the left and right lobes of the LAL appear to phasically inhibit contralateral output neurons (“flip-flopping,” Iwano et al., 2010). This actively increases the sampling of the sensory world and is seen for instance in the upregulation of scanning in ants that experience visual uncertainty (Wystrach et al., 2014).

CPGs have been shown to be useful components for motor systems in bio-inspired robots that mimic well-studied motor circuits such as the walking CPGs of the stick insect (Mantziaris et al., 2020) or salamander (Ijspeert, 2008). CPGs are neural circuits that produce rhythmic outputs in the absence of rhythmic input. Possibly the simplest CPG to imagine is the so-called half-center oscillator consisting of two inhibitory, adapting neurons (Daun et al., 2007; Shpiro et al., 2007). If one neuron is firing faster, it will inhibit the other neuron. While firing, it will adapt, its firing rate will decline, the other neuron is eventually released from inhibition and will resume firing. This leads to an even faster decline of the firing rate of the first neuron, and the process commences again with swapped roles for the neurons. The back and forth between the two CPG neurons leads to a phasically changing output. The characteristics of this CPG depend on the intrinsic properties of the neurons, in particular the spike rate adaptation.

Computational models of the LAL have been developed to obtain a better understanding of how the LAL network's “flip-flop” activity might be generated. One of the first attempts used genetic algorithms to generate a model that produces a flip-flop activity and has approximately the same connectivity as observed in experiments (Chiba et al., 2010). A recently developed rate-based model (Adden et al., 2020) implements a suggested LAL-network connectivity from the silkmoth (Mishima and Kanzaki, 1999). The switch mechanism was hard-coded and inspired by neurons switching between low- and high-firing states. In simulations, the model reproduced aspects of silkmoth behavior as shown by Ando et al. (2013), where mounted silkmoths on a robot followed pheromone plume edges. None of these models used a biologically plausible mechanism to reproduce the flip-flop activity.

In other modeling work the function of the LAL has been considered within larger models of the Central Complex of the insect brain, which is a brain area involved in control or orientation and heading (Pfeiffer and Homberg, 2014). The central complex is involved in path integration (PI) and recent models of PI use a simple representation of the LAL, which consists of a left and a right turning neuron (Stone et al., 2017; Sun et al., 2020) to integrate outputs of the PI network. Modeling of another Central Complex brain area called the ellipsoid body explored motor control learning. It provided a comprehensive account of the pre-motor role of the LAL by simply assigning the individual neurons of the LAL to different actions like forward, left and right turning commands (Fiore et al., 2017). Furthermore, a mechanistically similar model showed how using a CPG may explain continuous lateral oscillations as a core mechanism for taxis in *Drosphila* larvae (Wystrach et al., 2016). Thus, across a range of models the functionality of the LAL and its connection to behavior has been explored. Here, we build on this work to produce a biologically plausible model, that captures the general behavioral utility of the LAL pre-motor circuit.

**Figure 1:**
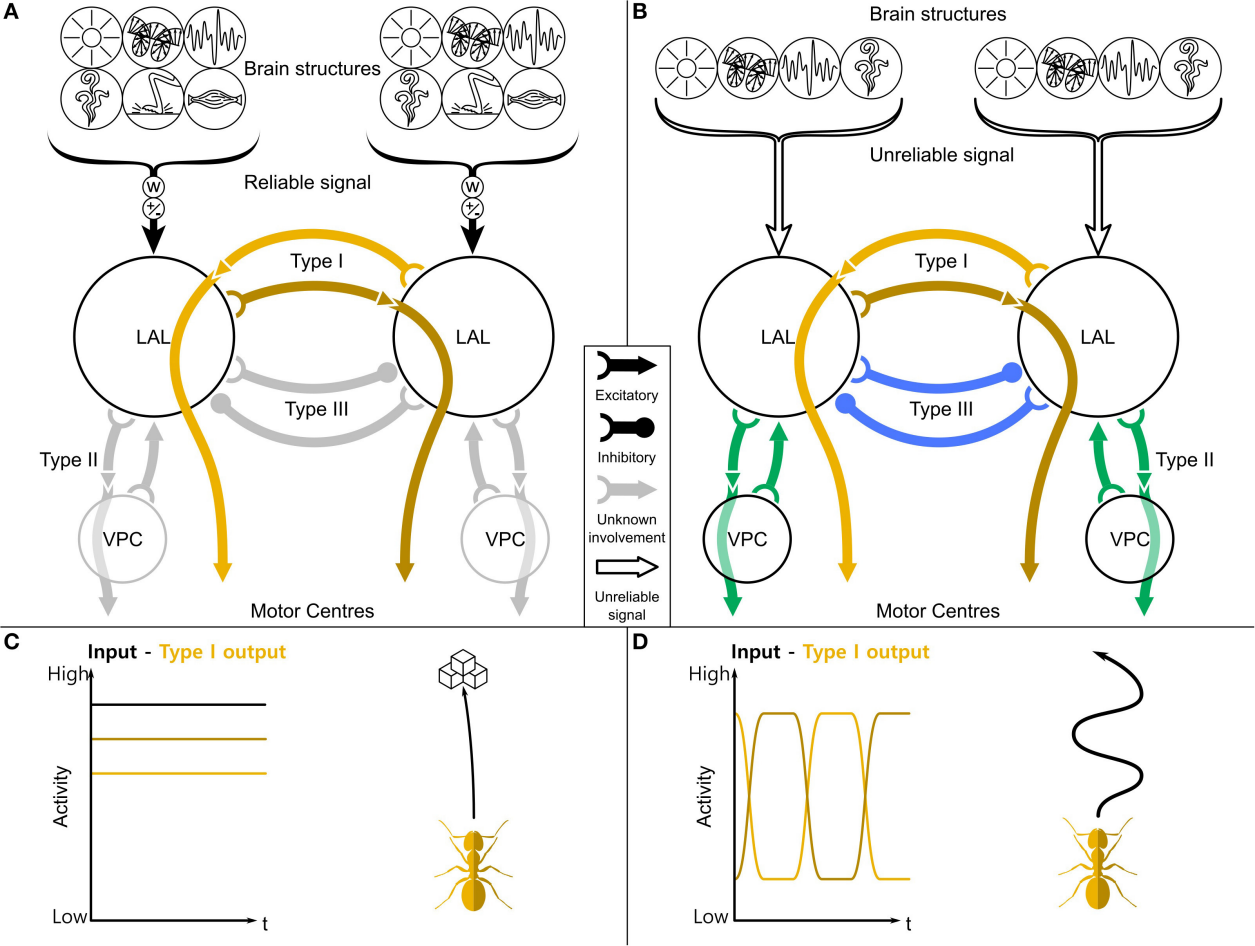
A general purpose steering framework based on the LAL. **(A)** The two LAL compartments receive inputs from many sensory modalities from the ipsilateral hemisphere, both low level sensory information (like optic flow, haptic, or olfactory signals) and highly processed information (path integration, place recognition). These inputs are differently weighted (w) and contain approach/aversion commands (±). If the receiving information is reliable (filled brackets), the turning signal will be directly gated through to the motor centers **(C)**. **(B)** If it is not reliable (empty brackets), a central pattern generator phasically flips the activity of the left and right output neurons. This leads to a search motif **(D)** and therefore to a higher sampling of the environment. While contralaterally descending neurons (Type I, orange) are mostly involved in steering, ipsilaterally descending neurons (Type II, green) and contralaterally inhibitory neurons (Type III, blue) are involved in the switching. LAL, lateral accessory lobe; VPC, ventral protocerebrum. Schematic based on Steinbeck et al. (2020a,b).

We have recently developed a general steering framework (see Figure 1, Steinbeck et al., 2020a), in which we showed how the conserved circuitry of the LAL and its connections is an excellent candidate for producing both targeted steering and oscillating search behaviors across a wide ranges of insect behaviors utilizing multiple sensory modalities. Based on this framework we here present spiking neural network models inspired by the LAL. We have constructed a Comprehensive model to explore the implications of diverse neurophysiological and neuroanatomical data on the LAL region and its descending neurons, to which we will refer to as the Comprehensive Network. We then further distilled a Core network with a focus on extracting the core mechanism underlying the principles of the steering framework, to which we will refer to as the Core Network. The exact network connectivity within the LAL is currently unknown. Therefore, we based our models' connectivity on both the known global anatomy and our hypothesis about how the observed output and correlated behaviors arise in the LAL. We explore how lateralized sensory input and lateralized motor circuits can integrate with a sensory modulated CPG to generate: (1) targeted steering for approaching a goal in response to clear sensory information; and (2) the generation of rhythmic output that can drive small scale searching patterns in the absence of reliable sensory information. Our aim is to demonstrate that the models can drive both these distinct behavioral modes solely from the interaction of sensory input with intrinsic network dynamics.

We explore the properties of our networks by situating them in a simple simulated animat. The Comprehensive model demonstrates that the adaptive output can be created from a network with specific neuron roles that are hypothesized to be close to their biological functions (Ibbotson, 1991; Bidwell and Goodman, 1993; Goodman et al., 2002; Schnell et al., 2017; Namiki et al., 2018; Bidaye et al., 2020; Rayshubskiy et al., 2020). In the Core network we explore a wide range of parameter combinations and show how this network can robustly generate an adaptive range of movement behaviors.

## 2. Models and methods

### 2.1. Implementation

Modeling and analysis were performed with MatLab 2017 & 2019 (The MathWorks, Inc., Natick, Massachusetts, USA). In addition to the core Matlab software we also used the Psychophysics Toolbox and the Statistics and Machine Learning Toolbox. Simulations were run on a Dell Latitude 5480 with Windows 10. The code to run the simulations and analysis as described in the methods can be found online at https://github.com/FabianSteinbeck/SNN-LAL-CPG.

### 2.2. Central pattern generator computational model

We have formulated the model as a spiking neural network consisting of leaky integrate-and-fire neurons with adaptation,


(1) $$\begin{array}{l} \frac{dV}{dt}=\frac{1}{C_{m}}(g_{\text{leak}}(V_{\text{leak}}-V)+g_{\text{adapt}}A^{P}(V_{\text{adapt}}-V)\\ \;+I_{0}+I_{syn})(1+\eta ) \end{array}$$

where $C_{m}=0.5\cdot 10^{-9}$F is the membrane capacitance, $g_{\text{leak}}=5\cdot 10^{-9}$S the leak conductance and *V*_leak_ = −60 mV the leak reversal potential. Once the voltage surpasses a threshold *V*_th_ = −50 mV, a spike is emitted, the membrane potential clamped to *V*_spike_ = 20 mV for the one timestep, and then to *V*_reset_ = −65 mV for a refractory period *t*_refract_ = 1 ms. The adaptation current is activated by spikes, *A*(*t*+Δ*t*) = *A*+Δ*A* (see Tables 1, 2 for values of Δ A), and decays exponentially,


(2) $$\begin{array}{l} \frac{dA}{dt}=-\frac{A}{\tau_{\text{adapt}}}. \end{array}$$

**Table 1:** Core network.

***w_EI_, w_EO_*, *w*_*II*_, *w*_*IO*_**	***g*_*adapt*_[S]**	***ΔA* [unitless]**	***p* [unitless]**	**τ_*adapt*_[s]**
0.5	0.25·10^−7^	0.01	1	0.05
1	0.5·10^−7^	0.05	1.5	0.1
2	1·10^−7^	0.1	2	0.2
3	2·10^−7^	0.2	3	0.3
4	4·10^−7^	0.5	4	0.5

Parameter values used in parameter space exploration.

**Table 2:** Core network.

**E[A]**	**I[A]**	**O[A]**
0	0	3.7698·10^−10^

Constant current offset values for different neuron types.

The adaptation current has conductance *g*_adapt_, time scale τ_adapt_, activation exponent *p* (see Tables 1, 2), and reversal potential *V*_adapt_ = −70 mV. The term 1+η implements multiplicative noise, where η is a uniformly distributed random variable, $\eta =3\cdot 10^{-6}rand\left(1\right)/\sqrt{dt}$. *I*_0_ is a constant current offset that sets the spontaneous activity levels of the neurons (see Tables 3, 4). The synaptic current *I*_syn_ is modeled with conductance based synapses,


(3) $$\begin{array}{l} I_{\text{syn}}=g_{\text{syn}}S\left(V_{\text{rev}}-V\right) \end{array}$$

**Table 3:** Comprehensive network.

***w*_*AI*_**	***g*_*adapt*_[S]**	***p* [unitless]**	***ΔA* [unitless]**	**τ_*adapt*_[s]**
−7	0.25·10^−7^	0.01	1	0.05
−8	0.5·10^−7^	0.05	2	0.1
−9	1·10^−7^	0.1	3	0.2
−10	2·10^−7^	0.2	4	0.3
−11	4·10^−7^	0.5	5	0.5
−12				

Parameter values used in parameter space exploration.

**Table 4:** Comprehensive network.

**E[A]**	**V[A]**	**IL[A]**	**CL[A]**	**A[A]**	**I[A]**
0	1.22510·10^−10^	5.5125·10^−11^	1.05·10^−10^	0	0

Constant current offset values for different neuron types.

where *g*_syn_ is the maximal synaptic conductance and *V*_rev_ = 0mV the reversal potential for excitatory and *V*_rev_ = −80mV for inhibitory synapses. The activation variable is incremented *S*(*t*+*t*) = *S*(*t*)+0.1 for every presynaptic spike and decays according to


(4) $$\begin{array}{l} \frac{dS}{dt}=-\frac{S}{\tau_{\text{syn}}} \end{array}$$

with timescale τ_syn_ex_ = 20 ms and τ_syn_in_ = 30 ms. The model was integrated with a forward Euler algorithm with constant time step Δ*t* = 1ms. The sensory inputs were generated with the same time resolution.

### 2.3. Design logic of the comprehensive network

**Figure 2:**
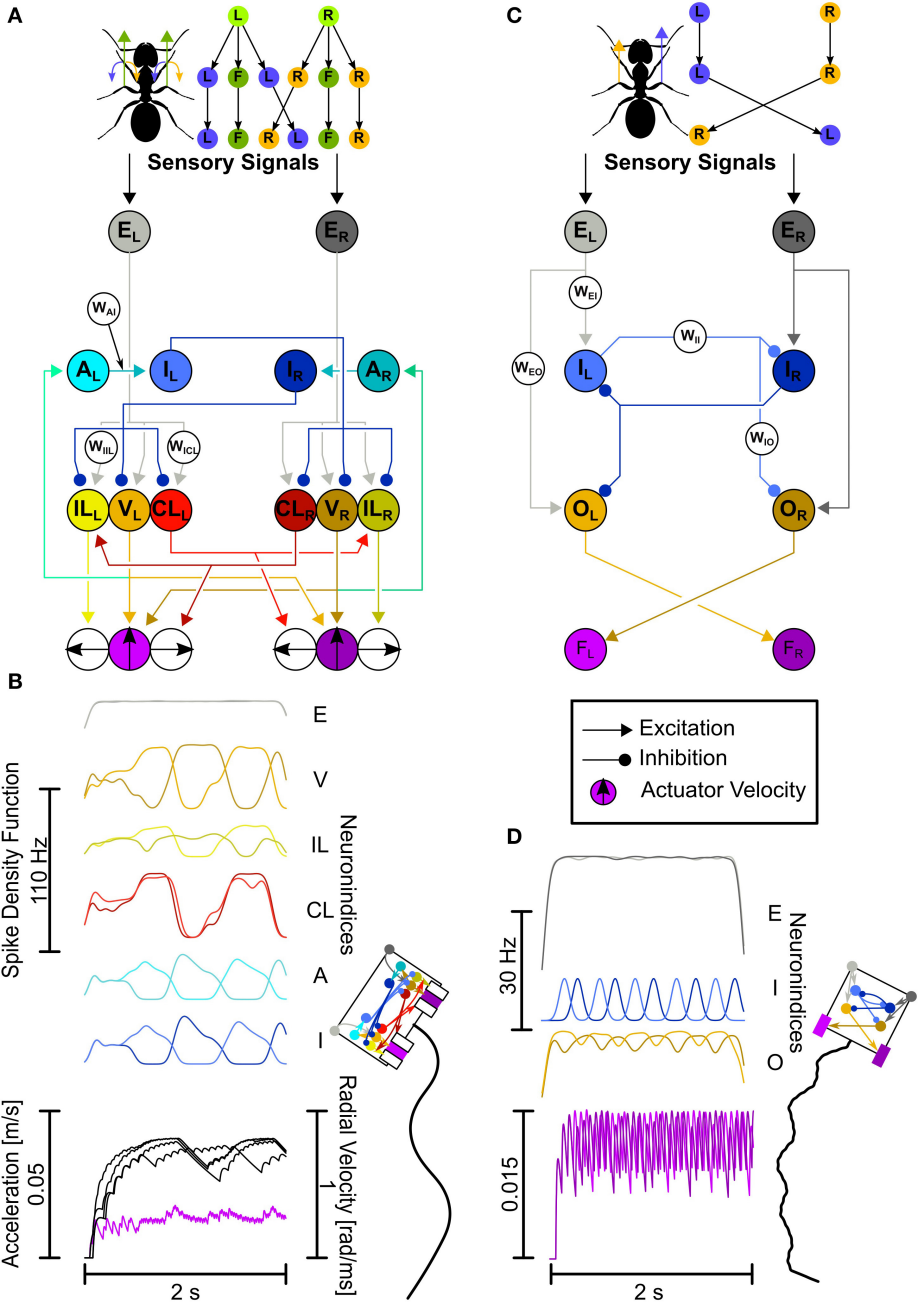
The LAL-inspired SNNs. **(A,B)** Comprehensive network. **(A)** Top: Motor control split into velocity and turning. L, left turn; R, right turn; F, forward. Bottom: This network explores connectivity and neuron functions as described in the text with dedicated velocity and rotation neurons, leading to Ackerman steering. Neuron Abbreviations: E, excitatory input; V, velocity; IL, ipsilateral turning; CL, contralateral turning; A, adaptation; I, inhibition. **(B)** Network activity plotted as Spike Density Functions (SDFs). Visual representation of the animat (not the actual actuator dimensions), and the trajectory generated with the activity depicted in **(B)**. **(C,D)** Each letter shows the same as in **(A,B)**, but for the Core network. Both the CPG circuit neurons and descending neurons have been unified. Neuron Abbreviations: E, excitatory input; I, adaptory inhibition; O, Output; F, force integrators. Colors for neurons: Gray, input; Orange, velocity/output; Yellow, ipsilateral turning; Red, contralateral turning; Cyan, adaptation; Blue, inhibition/adaptory inhibition; Purple, velocity actuator. Brighter colors, left hemisphere; darker colors, right hemisphere.

The overall level of input to the networks is coding for the reliability of a not further specified sensory signal and enters into the input neurons (Figures 2A,C) through static input currents in the range (0, 1.75·10^−9^A). High overall input current means a highly reliable signal and vice versa, similar as in basis function network modeling (Deneve et al., 2001). The balance of input on the left and right carries information about whether to go left or right, regardless of the overall signal strength. The networks here are designed so that the left hemisphere controls left turns and the right hemisphere controls right turns, as information has been shown to be processed unilaterally (Paulk et al., 2015), similarly to a Braitenberg vehicle (Braitenberg, 1984). Therefore, when receiving unilateral input, the SNNs are intended to generate a proportional unilateral output.

When the inputs are bilateral, the circuits should generate a flip-flopping output. Furthermore, weak sensory input should result in slow flip-flopping (leading to large exploratory turns), whereas strong activation of the CPG results in faster flip-flopping (leading to straight movements with small undulation). The outputs can then be used to directly generate movement (see Figure 2C).

The Comprehensive network explores neurophysiological and neuroanatomical aspects in more detail. The two aspects explored here are: (1) the motor control is split into velocity (“V”-neurons) and rotation neurons (“IL”-neurons and “CL”-neurons, Figure 2A), and (2) the CPG is created by adapting (“A”-neurons) and inhibitory neurons (“I”-neurons, Figure 2A). Unilaterally descending velocity neurons (V) are connecting to motor centers on both sides for velocity control and connect to the ipsilateral adapting neurons (A). The adapting neurons connect to the ipsilateral inhibitory neurons (I), which in turn connect to the contralateral velocity (V) and rotation neurons (IL, CL). This leads to a CPG with a few extra steps (see Supplementary Figure 1). Ipsilaterally descending rotation neurons (IL) connect to the ipsilateral motor center initiating ipsilateral turns, i.e., neurons located on the left side descend to the left side of the motor centers and induce left-ward turns. Contralaterally descending rotation neurons (CL) connect to the contralateral motor center initiating ipsilateral turns, i.e., neurons originating in the left-hemisphere descend to the right side of the motor centers and induce left-ward turns. The CL neurons additionally connect to contralaterally located IL neurons.

The actuators of the animat work by directional force generation. As real legs can generate forces independently into all directions, our representation here aims for the same, excluding backward motion. While the forward facing actuators only control the forward movement velocity, the sideward facing actuators generate movements laterally. Each actuator group, the left and the right, ultimately generate a single force vector, which is the result of each independent vector added onto each other. Since the V-neurons connect to both forward movement actuators, the forward force will be the same for both actuators. Activating the same sideward actuators with the same strength, i.e., the left-facing actuators by exciting the *IL*_*L*_ and *CL*_*L*_ neurons, this would result in a leftward translation. In order to be able to rotate toward the left, the leftward force on both actuator sides should be different in strength. This is achieved with the CL neurons additionally connecting to the IL neurons contralaterally, in the leftward rotation case with the *CL*_*L*_ neuron additionally connecting to the *IL*_*R*_ neuron. This leads to a lesser leftward turning command of the outside actuators (the right group) than the inside actuators (the left group), because the leftward command is lessened by the additional rightward command - the outside turning radius becomes lesser than the inside turning radius, similarly to Ackermann steering. Ackermann steering is a geometric arrangement of linkages in steering such that the actuators on the outside follow a wider radius than the inside actuators. An example would be steering a car, where, if the wheels would be statically linked, both the inside and outside wheels would follow the same radius of turning, which leads to inefficiencies. Instead, by adding dynamic linkages, the wheels can follow slightly different radii, adapting them to the actual turn.

Putting this all together should lead to a desired behavior: when the network receives differently strong inputs from left and right, it should generate a steering response, e.g., if the left input is stronger than the right input, it should steer leftwards. When it receives similarly strong inputs, it should generate a zig-zagging behavior. Furthermore, when the strength of the similar inputs are relatively weak, the zig-zagging response should be more exploratory, and when the inputs are strong, the zig-zagging response should be less exploratory and overall forward.

Weak and strong in this context are relative terms, but also can be grounded in terms of frequency ranges, as neurons display lower ranges and higher ranges of activity; many neurons within this brain region fire around 15–25 Hz when not active, and between 50–100 Hz when active. Therefore we stayed within these boundaries (Steinbeck et al., 2020a).

### 2.4. Design logic of the core network

The base layout of the Core network builds on the key aspects of the Comprehensive network, namely the hemispheric division and the usage of a CPG. However, we combined and simplified the CPG circuit and the motor control (see Figure 2D). The computational algorithm is the same. Here, input neurons (“E”-neurons) make ipsilateral, excitatory connections to the CPG neurons (“I”-neurons), and output neurons (“O”-neurons). Each of the two CPG neurons provides inhibitory input to the contralateral CPG neuron and output neuron. Additionally, the inhibitory neurons are themselves adapting. The excitatory output neurons, which have been parameterized to produce a spontaneous spike rate of circa 30 Hz (corresponding to the mean of rates reported in the literature; Iwano et al., 2010; Zorović and Hedwig, 2013; Namiki and Kanzaki, 2016) connect to the contralateral force integrators.

The desired behavior is the same for the Core-Network as for the Comprehensive network.

### 2.5. Embodiment

The actuators are modeled as non-spiking integrators. The maximum value they can achieve with prolonged excitation (spike trains) represents the maximum velocity each actuator can generate. Non-spiking integrators approximate computational models of muscles (Wexler et al., 1997).

The actuator dimensions and movement values used are inspired by movements of *Melophorus bagoti* (Wystrach et al., 2014) and the actuators are arranged as in a Braitenberg vehicle (Figures 2B,D). Each actuator generates forward velocity independently of the other. In the Core network, if the right actuator generates a higher velocity, the agent will turn left by rotating around the body center point, and vice versa.

In the Comprehensive network, the forward motion and rotation are calculated independently from each other (Figure 2A, top); both actuator sides generate leftward and rightward turning values, which are averaged for each actuator side first, then averaged over both actuators. The forward motion is the same for both actuators. As described in the previous section, due to the connectivity, this will produce Ackermann steering.

### 2.6. Parameter space exploration

The values chosen for the networks were in the typical range of parameters within which neurons operate. Within these ranges, we first ran simulations to find combinations that would generate flip-flopping. Then to find further combinations, we expand the parameter space outwards from previously successful values.

#### 2.6.1. Comprehensive network

The parameter exploration focused on finding suitable parameters to generate the desired behavior in order to validate that the model can produce the two adaptive motor patterns that the LAL is known to produce. Thus we explored a focused region of the model's parameter space, related to CPG dynamics, namely the parameters of the adaptation current (the five parameters as shown in Table 2). This resulted in 5^4^·6 = 3,750 simulations.

In the second phase we focused on demonstrating that a parameter combination existed that could additionally generate reasonable steering behavior, in addition to the desired zig-zagging behavior. From the explored phenotypes of the first phase we used the 34 phenotypes (within this paper we define the genotype as the combination of the possible parameters, and the phenotype as the resulting movement pattern, which a particular parameter combination generates), which produced the most desirable CPG behaviors in the first phase, which is the increase of the speed of flip-flops with increased symmetrically strong inputs. Therefore, ultimately, in the Comprehensive network we only explored different weights from the inhibitory neurons to the contralateral descending turning neurons (*w*_*IIL*_, *w*_*ICL*_), tuning for the desired rotation behavior. The 10 weights explored were −1, −2.6, −4.1, −5.7, −7.2, −8.8, −10.3, −11.9, −13.4, −15, leading to 100 simulations per phenotype, resulting in 10·100·34 = 3,400 simulations.

#### 2.6.2. Core network

We simulated the model for 2,000 timesteps modifying four adaptation related neuron parameters and four weights, *w*_*EI*_, *w*_*EO*_, *w*_*II*_, and *w*_*IO*_, each with five values (see Table 1). Each genotype was run five times, where the input strengths were given at a constant 25, 50, 75, and 100% from both inputs, and an asymmetrical combination of 25% left input and 100% right input (which should result in a right turn), resulting in 5^8^ = 390,625 simulations.

Unsuccessful simulations, as defined by the following rules, were excluded:

1. The CPG layer produced < 2 spikes (no flip-flopping with symmetric input) in both the Core and Comprehensive version
2. The maximal spike rate of output neurons was >60 spikes/s (Core) or 120 spikes/s (Comprehensive)

If, according to these rules, any of the 5 simulations for a given genotype were unsuccessful, the whole genotype was excluded.

### 2.7. Trajectory analysis

**Figure 3:**
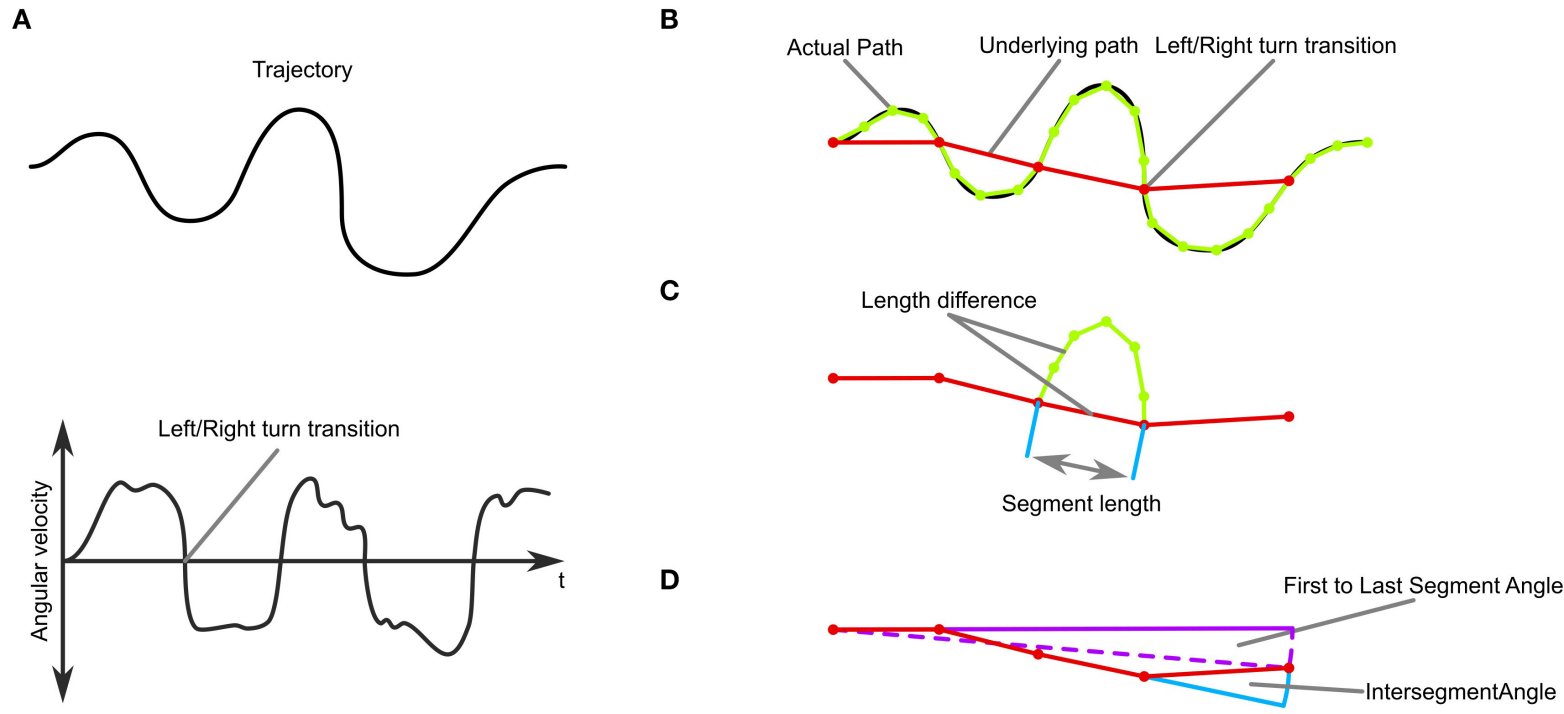
Trajectory-analysis. **(A)** Top: A simulated trajectory. Bottom: The trajectory's angular velocity profile. **(B)** An underlying path, where each segment connects to the positions of left and right turning transitions. **(C)** Measures of sinuosity: the length difference between the path and the underlying path in each segment, the number of segments, and the length of each segment. **(D)** Measures of stability: Overall movement direction and intersegment angle.

We were mostly interested in the behavioral outcome of the CPG dynamics and focussed the analysis on the zig-zag quality (frequent and evenly spaced changes of steering direction) of the agents' trajectories. We subdivided the trajectory into segments by identifying the transition points from left turns to right turns and vice versa (the points where the angular velocity changed sign, Figures 3A,B). We defined an “underlying trajectory” by connecting these points with straight line segments. Turns where the angular velocity was smaller than 1·10^−3^rad/ms were regarded as low-level noise and excluded from this analysis.

The segmentation and definition of the underlying trajectory then enabled us to analyse the stability and sinuosity of the trajectory (Figures 3C,D). As a measure of *sinuosity* we used the length difference between the actual trajectory and the underlying trajectory. The bigger the difference, the curvier the actual trajectory is between transitions. Additionally, we used the number of transitions per trial, as well as the length of the segments (Figure 3C). We defined trajectories as *stable* when they did not change in heading over time as judged by the angle between the first and last segment of the underlying trajectory (Figure 3D). Additionally, we analyzed the inter-segment angles and the origin to final position angle as a measure of consistency of movement direction.

#### 2.7.1. Comprehensive network

In the first phase we focused solely on the zig-zagging dynamics, in this case on the increase of the number of switches with the input amplitude. In the second phase we focused on the steering dynamics, where we explored inhibitory neurons to rotation neuron weights, we analyzed all 3,400 phenotypes visually.

#### 2.7.2. Core network

We are interested in a phenotype where small inputs lead to exploratory behavior and large inputs to goal directed behavior. We therefore restricted the search by the following conditions, which we deemed to increase the chances of identifying models with sensible phenotypes we are searching for:

1. Sinuosity should decrease with input amplitude: The number of transitions should increase and the length of the segments should decrease; or transitions should disappear altogether.
2. Trajectories should be stable: The median intersegment angle should be within ±0.1 rad, the first to last segment angle within ±0.25 rad and the trajectory angle within ±0.25 rad.

## 3. Results

We implemented two models of the LAL network, a Comprehensive model that includes all of the known cell types and anatomical connections and a Core model where neuron types are combined into functional groups. For both models we investigated how the CPG dynamics lead to specific types of movement trajectories, depending on the parameters of neurons and synapses in the model. We then asked, what kind of parameter combinations led to movement trajectories where small sensory inputs lead to exploration and large inputs to goal directed movement.

### 3.1. Comprehensive network

Our initial aim was to develop a SNN model that incorporates current knowledge of LAL neuron types and putative functions (Steinbeck et al., 2020a). The resultant Comprehensive model (see Figure 2A) contains 12 neurons and 35 parameters, out of which we investigated seven parameters. The model consists of two functional groups, motor control neurons, and CPG neurons.

The input neurons (E) feed into the motor control neurons, which in turn feed into the ipsilateral actuator (IL for turning, V for velocity) and the contralateral actuator (CL for turning). The velocity neurons furthermore feed into the CPG neuron pathway; Supplementary Figure 1 illustrates the CPG components, which lead to the rythmic inhibition and therefore motif generation.

**Figure 4:**
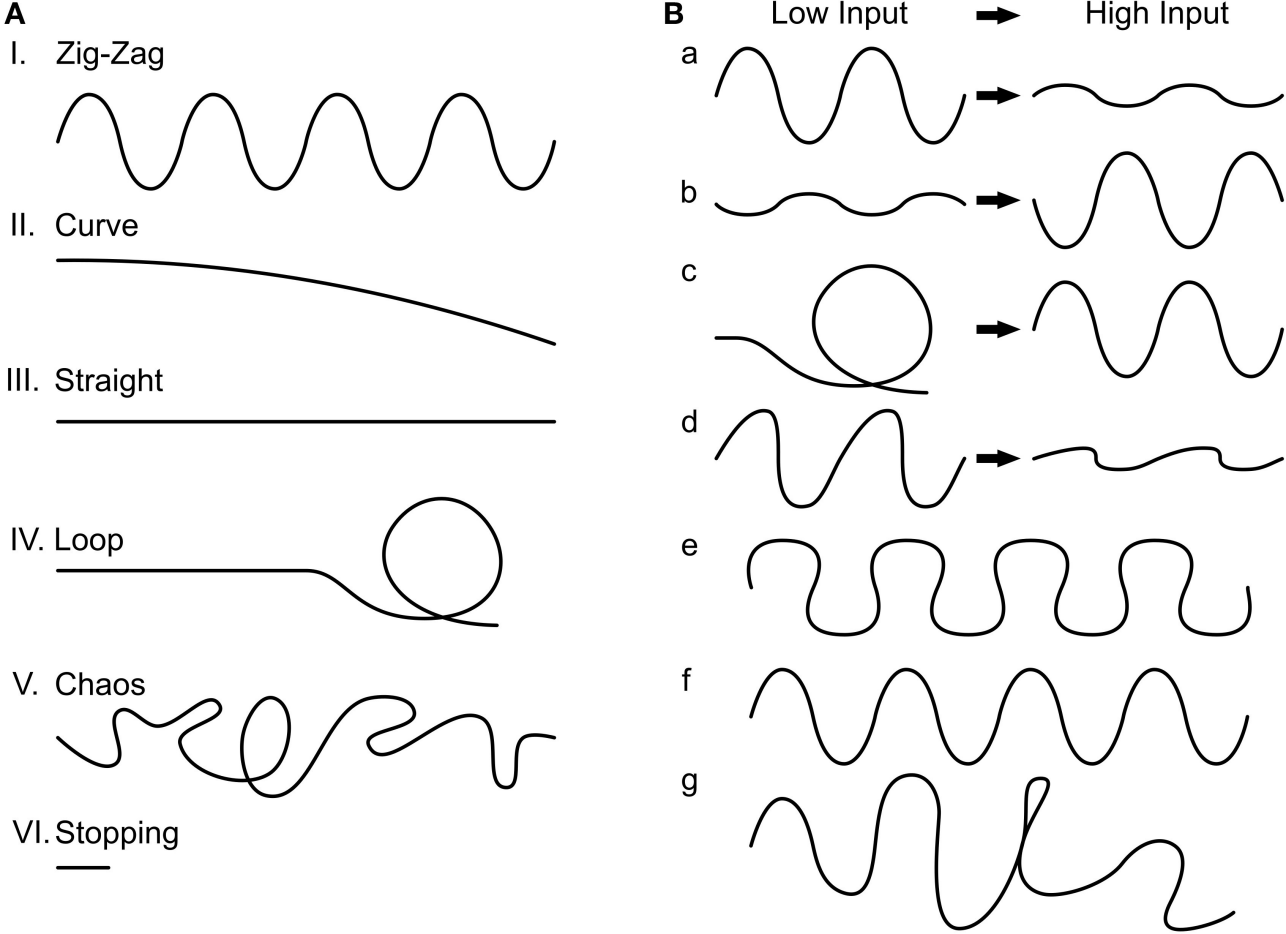
Trajectory phenotypes. **(A)** We found six common phenotypes. I. Zig-zag, II. Curve, III. Straight, IV. Loop, V. Chaos, and VI. Stopping. **(B)** The zig-zag phenotype we further divided into seven subcategories. a: The change in trajectory from low input to high input is reflected in less pronounced zig-zagging (desired), b: the reverse, c: where the agent loops with low input but zig-zags with high input, d: asymmetrical zig-zagging (which behaves as a) otherwise, e: curvaceous zig-zagging, f: just zig-zagging, and g: noisy zig-zagging.

Characterization of the final 3,400 genotypes showed the following phenotype distributions: zig-zag (86.47%), straight (11.44%), curve (1.12%), and chaos (0.88%). Within zig-zagging, we further divided seven sub-phenotypes (Figure 4B, percentages scale to 2,761 zig-zag phenotypes): curvier with low input to straighter with high input zig-zagging (34.63%), straighter to curvier zig-zagging (4.69%), curvaceous zig-zagging (19.52%), curve to zig-zagging (0.68%), just zig-zagging (7.24%), and randomly occurring zig-zagging (33.23%). The simulations with asymmetric input (see Section Materials and methods) always resulted in a deterministic rotation which reflects the difference in inputs on the two sides.

**Figure 5:**
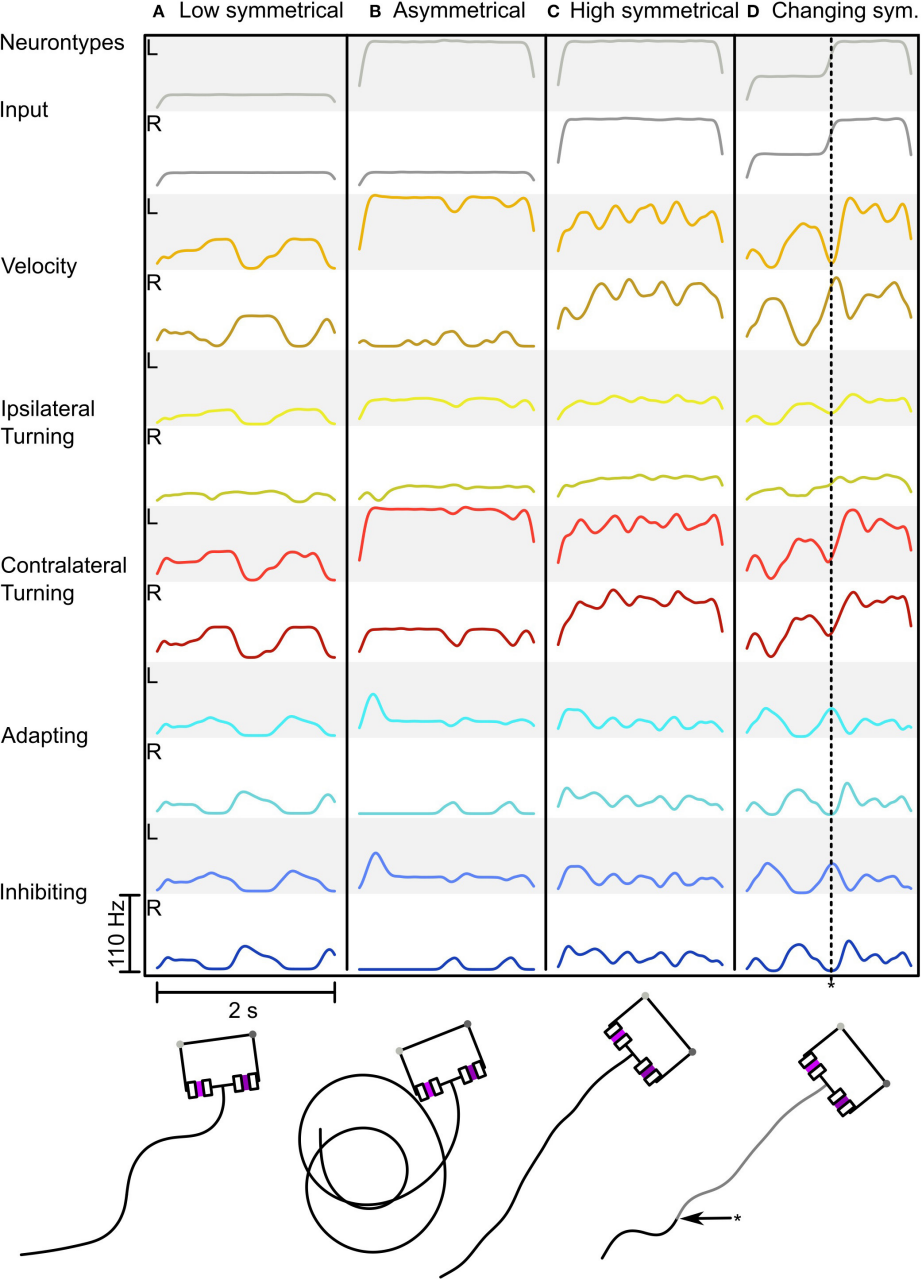
Behavior of the Comprehensive network in response to different sensory inputs. **(A–D)** Shows the network activity as a Spike Density Function (SDF) during a simulation run with the resulting trajectory below (for neuron types see Figure 2). **(A)** Low symmetrical input leading to strong zig-zagging. **(B)** Asymmetrical input leading to circling. **(C)** High symmetrical input leading to minimal zig-zagging with forward motion. **(D)** Changing input from medium to high symmetrical input (asterisk * indicating the time point of input change in both the neural trace and in the motion trace) leading to firstly zig-zagging to forward motion. The SDF used a Gaussian Kernel with μ = 0 and σ = 0.05 over 500 ms.

Figure 5 shows the adaptive motor patterns that are produced by one parameter combination for a range of sensory inputs. It was selected because it looks like the behavior we desire. The combination: *w*_*AI*_ = -2.5, *w*_*IIL*_ = -12, *w*_*ICL*_ = -8.8, *g*_*adapt*_ = 4·10^−7^S,p = 0.05, Δ*A* = 4, τ_*adapt*_ = 0.5s, produces oscillatory behavior with weak inputs (see Figures 5A,D) and strong sensory signals produce turning when asymmetrical (Figure 5B) or fast straight movement when balanced (see Figure 5C). Thus we have a proof of concept that the Comprehensive network can function adaptively (in the sense of dynamic motor control) as a steering network and as a CPG generating oscillatory search behavior.

### 3.2. Core network

We then tested the Core model network (Figure 2C), which contains six neurons and 30 parameters, out of which we investigated eight parameters. This network generates a variety of movement patterns depending on the parameters. Figure 2 shows the network producing the desired behavior, with a parameter combination of: *w*_*EI*_ = 0.5, *w*_*EO*_ = 0.5, *w*_*II*_ = −3, *w*_*IO*_ = −5, *g*_*adapt*_ = 2·10^−7^S, Δ*A* = 0.1, *p* = 3, τ_*adapt*_ = 0.5 s. Out of the 390,625 tested phenotypes, 249,624 did not meet the selection criteria (the CPG did not spike in each simulation or the output activity was too high, see Section Materials and methods). From the remaining 141,001 phenotypes, 1,580 met at least four out of six sinuosity and stability conditions, 200 met at least five out of six and 38 met at six out of six. We used these exclusion criteria with the logic, that if a trajectory with the desired properties is not generated when simulating once, it will not occur consistently and the phenotype can hence be excluded.

We visually inspected the generated trajectories in the two groups of selected phenotypes. From the six typical trajectory phenotypes that can generally be identified (Figure 4A), we encountered four within the inspected phenotypes (percentages are with respect to the 1,580 inspected phenotypes): “zig-zagging” (66.39%), “straight” (33.16%), and “curved” (0.003%). Within zig-zagging, we further divided seven sub-phenotypes (Figure 4B), percentages are relative to the 1,049 zig-zag phenotypes: curvier to straighter (for low to high input) zig-zagging (60.91%), straighter to curvier zig-zagging (0.16%), asymmetric zig-zagging (5.81%), just zig-zagging (27.55%), and randomly occurring zig-zagging (4.1%). The asymmetrical input simulations always resulted in a deterministic rotation. Thus, we have shown that the network can generate a variety of movement patterns by the use of different parameters; specifically, many parameter combinations lead to the desired zig-zagging behavior.

### 3.3. The network is robust to parameter changes

We ran a principal component analysis (PCA) on the successful genotypes, specifically on the systematically explored parameters. By this we investigated whether there was a lower dimensional subspace of parameter combination that led to successful phenotypes and, in particular which parameter combinations mostly contribute to the zig-zagging phenotype. We used Min-Max scaling or standardization to preprocess the parameters. We ran PCAs on the set of all parameter combinations that 1. produced trajectories at all (~140,000), 2. produced “sensible” trajectories (~1,600, which are the ones which the trajectory analysis narrowed down for inspection), 3. produced interesting zig-zagging trajectories (~1,500, which are including the phenotypes I [a, d, f], II, III and are a subset of the sensible trajectories), and 4. produced the best zig-zagging trajectories (~1,000, which are including the phenotypes I [a, d, f] and are a subset of the interesting trajectories).

In all cases the variance explained by the first principal components was low. In particular, the first principal component (PC1) for the best trajectories, PC1 explained only 17% of the variance, for all other trajectories even less. The leads us to believe, that the zig-zagging phenotype can occur with many different genotypes of network parameters. We compared different parameter combinations to investigate how big the spread of the best zig-zagging is (Supplementary Figure 2). Most combinations yield a big spread, but some parameter combinations are more likely to produce the desired behavior.

**Figure 6:**
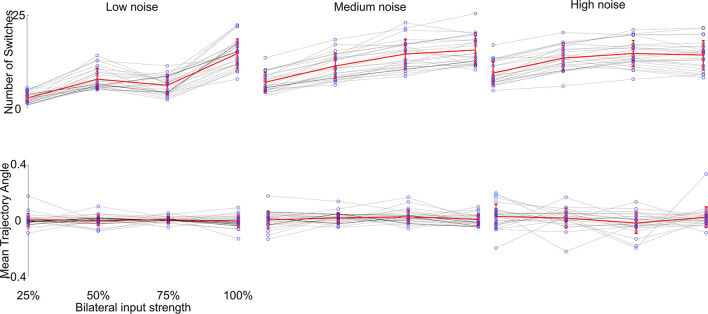
Reproducibility of the desired behavior (stronger zig-zagging with weak input, weaker zig-zagging with stronger input) of the Core network with different levels of noise. We chose 25 parameter combinations which showed great approximations of the desired behavior (see Figure 4Ba) and changed the noise component η of the voltage computations (see Equation 1). Each behavior was simulated 100 times. The top row shows the number of switches per simulation, the bottom row the mean trajectory angle (the overall trajectory direction from start to end) with different symmetric input strengths (gray lines and blue dots) over 100 simulations. The overall behavior (mean of all means in red) stayed the same with increased switching from increased input strength, while the variability increased with increased noise level as expected.

We then investigated the reproducibility of the generated trajectories, since the simulations were performed only once per parameter combination. We selected visually the phenotypes which produced the desired behavior best. We repeated each of these 100 times and compared the resulting behavior. The only term influencing the outcome of each single parameter combination simulation is the additional noise component during the neuronal computation (η in Equation 1). This would result in a slightly different trajectory with each simulation. To further investigate the influence of the noise component, we changed the noise amplitude to 5-fold higher and 10-fold lower than the original value and compared the generated trajectories, see Figure 6. We found that the noise component largely determines the overall outcome of the variability of trajectories, which seems to be determined by the phase building up to the flip-flopping behavior (see Figure 2D). Yet, the overall zig-zagging behavior typically stays the same, meaning that zig-zags continue to get faster with higher input and the overall forward motion remains similar. Thus, we have shown that the generated behavior is robustly generated with the Core network.

## 4. Discussion

### 4.1. Takehome message

We set out to explore how a neural network could produce adaptive behavior in a navigation setting. We built the spiking neural networks based on the overarching layout of the LAL and the behaviors it should produce (Steinbeck et al., 2020a). We chose the networks to keep a hemispheric layout, while simultaneously containing a CPG. A non-symmetric input resulted in a non-symmetric output, while symmetric input generated searching behavior for lower input strength and forward movement for higher input strength. Therefore, if a reliable stimulus appeared toward one side, the agent would steer toward it, whereas if it appeared ahead, the agent would approach it directly, similarly to as phonotaxis in crickets (*Gryllus bimaculatus*, Zorović and Hedwig, 2013). If the sought desired stimulus could not be perceived though, the agent would generate small scale search motifs, similarly to the zig-zagging in silkmoths (*Bombyx mori*, Kanzaki et al., 1992).

There are many different combinations of neuron parameters and connection weights leading to the desired behavior, even with varying amounts of noise, indicating that the network's setup may be well suited to generate this kind of behavior. The network was capable of generating a range of other natural seeming movement patterns for example the familiarity dependent zig-zagging trajectories of ants pursuing visual stimuli (Wystrach et al., 2014) or the pheromone plume pursuit of silkmoths (Iwano et al., 2010). This indicates that this network architecture can be easily adapted to generate different adaptive behaviors.

### 4.2. Flip-flop mechanisms

How do the inhibitory neurons switch between higher and lower firing rates? We choose the flip-flop mechanism to be an intrinsic adaptation, which is only driven by the reliability of a navigation relevant stimulus. This way, the mutual inhibition will lead to a CPG behavior and the flip-flopping will only occur if the neurons are activated simultaneously with a similar strength.

The bilaterally similar input strengths leading to a flip-flop behavior emerged in the network as described by Adden et al. (2020) as well. Their rate based model has morphological similarities to our model. However, the flip-flop driving mechanism is a hardcoded inhibition of spike rate, if the opposite population of neurons enters a high spike rate. This type of switching is inspired by bi-stable neurons which have low-firing states and high-firing states they can switch between (Gruber et al., 2003). Set into a navigation task, this model reproduces olfactory tracking behaviors and path integration behaviors.

In our Comprehensive network we investigate the interaction between the Ventral Protocerebrum (VPC) and LAL (Iwano et al., 2010). This bidirectional connection seems to be established by unilaterally descending and ascending neurons. The flip-flopping of Type I neurons may be a result of preceding network activity, and not being the neuron type which produces the flip-flopping by itself. This would make sense as Type I neurons directly project toward the motor centers. If Type I neurons were responsible for both initiating steering and flip-flopping, the species of insects (moths) which use a kind of zig-zagging, would zig-zag continuously to a stronger or lesser degree. While some species of ants seem to continuously zig-zag while navigating (Möel and Wystrach, 2020), silkmoths only flip-flop when tracking pheromone plumes. Therefore, we chose the adaptation neuron to be the ascending Type II neuron coming from the VPC, which innervates the inhibitory Type III neurons in the LAL, which in turn inhibit the contralateral Type I neurons.

In our Core network, flip-flopping occurs as a result of integrating the input neurons' activity and simultaneous mutual inhibition by Type III neurons, which are intrinsically adaptative. The build-up of the adaptation should be slow enough for the agent to actually be able to steer around for weak inputs, but fast enough to not steer much when receiving strong inputs. The power term *A*^*P*^ therefore may lead to fast build up, which is why mostly this behavior occurs with a low power term when compared with any τ_adapt_.

Other pathways may influence the flip-flopping mechanism in moths, one of them being optic flow (Pansopha et al., 2014). Moths alter their behavior, if the optic flow direction matches the expected direction, but the extent of perceived optic flow increases or decreases their turns. If the optic flow does not match, the moths perform a stereotyped pattern. Therefore, we suggest the adaptation mechanism to be partly be driven by external stimulus like optic flow. Incorporating optic flow to directly control the adaptation mechanism may increase spatial reliability of the CPG mechanism as it would be linked to external orientation stimuli. Furthermore, modulatory inputs to the LAL may alter the LAL's function depending on the agent's motivational state (Manjila et al., 2019). This could function as a “switch” to activate/deactivate specific functions within this network (maybe the CPG subnetwork) or deactivate the LAL as a whole.

### 4.3. What do the output neurons encode for?

Which kind of information is actually sent to the motor centers? In the Core network we simulated the outputs to the motor system to act similarly as a Braitenberg-vehicle, where we pick up on the observation that steering is initiated due to an imbalance of neuron activity between hemispheres (Iwano et al., 2010; Zorović and Hedwig, 2013). With this set-up, the actuation difference between left and right achieved should be high, so that the force generation difference is big enough to steer. Therefore, the connection from the inhibitory neurons to the output neurons (*w*_*IO*_) should be strong.

In the Comprehensive network we suggest a split into turning and velocity neurons due to optic flow integration in descending neurons in bees and the latest discoveries in the descending neurons of *Drosphila*. Descending neurons have been shown to innervate different groups of muscles: while some groups target muscle groups which are more involved in power generation, others target muscle groups which are more involved in steering (Namiki et al., 2018). Unilaterally descending neurons in *Drosphila* have been shown to code for velocity (Bidaye et al., 2020). Some of unilaterally descending neurons show bilateral innervation of the motor centers (Bidwell and Goodman, 1993). Functionally, this could control walking velocity across both sides of the motor centers. Other Type II neurons have been shown to be involved mostly in steering (Schnell et al., 2017; Rayshubskiy et al., 2020). Also, Type I neurons are involved in steering (Iwano et al., 2010; Zorović and Hedwig, 2013; Namiki and Kanzaki, 2016). Animals (insects) steer their bodies while walking by pushing the outward actuators away from the body while pulling in the actuators on the inside (Mantziaris et al., 2020). Both actuator centers therefore can produce both left and right turns individually. Fiore et al. (2017) suggested a similar pre-motor command structure (walking forward, left, and right in both LALs), yet more abstract than our Comprehensive model. The biggest difference to our models is the assumption that the LAL neurons are inhibited by CX neurons (Fiore et al., 2015).

However, evidence suggests that the meta-motor commands could be more nuanced than this. In cockroaches, recording of CX neurons show how different units encode for different walking directions and velocity (horizontal representation) (Martin et al., 2015). In flying insects like bees, different descending neurons are activated by different directions and velocities of optic flow, therefore coding for pitch, roll and yaw (vertical representation) (Ibbotson, 1991; Bidwell and Goodman, 1993; Goodman et al., 2002). This could mean that motor commands are encoded in a three-dimensional vector space, where a neuron encodes for a specific movement direction in an idiosyncratic manner, as well as the velocity into that direction. Therefore, motor command encoding could possibly be representing walking movements and flying movements separately, or when in either state, only a subset of all possible motor commands could be active.

Another question is how both the left and right descending commands are coordinated. While for a rotation an imbalance of activity between left and right may be sufficient to initiate rotational movements (steering), how are translational movements coordinated? We know from both walking and flying insects that these movements are used (Ravi et al., 2019). This would point toward an identical motor command representation in both pairs of the LAL, where both hemispheres encode omnidirectional movements and velocities, and the consequent motor group innervations are mirror symmetrical.

## 5. Conclusion

We have investigated the network dynamics of a spiking neural network model inspired by the Lateral Accessory Lobe of the insect brain. The network dynamics produce a stimulus reliability dependent small scale search behavior. That behavior can be produced over a variety of parameter combinations and noise levels, speaking of a robust network. Furthermore, other generated behaviors resemble naturally occurring behaviors of navigating animals.

In preliminary simulations we investigated the network in a simple navigation setting (Steinbeck et al., 2020b). We found that the generation of small scale search movements, which are directly modulated by the reliability of sensory signals, can improve the success of approaching a target. In future studies we want to incorporate this model with more complex visual stimuli (Risse et al., 2018), and additional modalities like optic flow (for the CPG adaptation mechanism Pansopha et al., 2014 or optomotor response Bidwell and Goodman, 1993) or olfaction (Ando et al., 2013) and navigation models (Möel and Wystrach, 2020; Sun et al., 2020).

## Data availability statement

The datasets presented in this study can be found in online repositories. The name of the repository and accession number can be found at: GitHub, https://github.com/FabianSteinbeck/SNN-LAL-CPG.

## Author contributions

FS developed the hypotheses, designed and programmed the neural networks, and performed the experiments and analysis. He furthermore wrote the manuscript. PG helped with experimental design and analysis, and editing of the manuscript. TN supported the design and development of the neural networks and editing the manuscript. AP supported with editing the manuscript. TN, AP, and PG acquired funding and developed the overarching research programme. All authors contributed to the article and approved the submitted version.

## Funding

FS was supported by a studentship from the School of Life Sciences, University of Sussex. PG, AP, and TN were supported by EPSRC (Brains on Board project, grant number EP/P006094/1 and active AI project, grant number EP/S030964/1). TN was also supported by the European Union's Horizon 2020 research and innovation program under Grant Agreements 785907 (HBP SGA2) and 945539 (HBP SGA3).

## Conflict of interest

The authors declare that the research was conducted in the absence of any commercial or financial relationships that could be construed as a potential conflict of interest.

## Publisher's note

All claims expressed in this article are solely those of the authors and do not necessarily represent those of their affiliated organizations, or those of the publisher, the editors and the reviewers. Any product that may be evaluated in this article, or claim that may be made by its manufacturer, is not guaranteed or endorsed by the publisher.

## Supplementary material

Supplementary Figure 1 Comprehensive network: Central Pattern Generator pathway. **(A)** The left-hand pathway (the colorful neurons and connections). The velocity neuron *V* drives the adapting neuron *A*, which in turn drives the inhibitory neuron *I*. *I*'s output then inhibits the contra-lateral descending neurons. **(B)** The right-hand pathway.

Supplementary Figure 2 Parameter combination relationships producing the desired behaviors in the Core network. Desired behavior: With low symmetric input the trajectory is strongly zig-zagging, with high input barely zig-zagging (see Figure 4Ba). The heatmaps show the network parameters plotted against each other in pairs. Some of the parameter pairs show clusters of combinations that are more likely to lead to the desired behavior. Many pairs show no clustering.
