# Supplementary figures and images for: Production of adaptive movement patterns via an insect inspired spiking neural network central pattern generator
Source: Front Comput Neurosci. 2022 Nov 18;16:948973. doi: 10.3389/fncom.2022.948973 (PMC9716565; doi:10.3389/fncom.2022.948973)

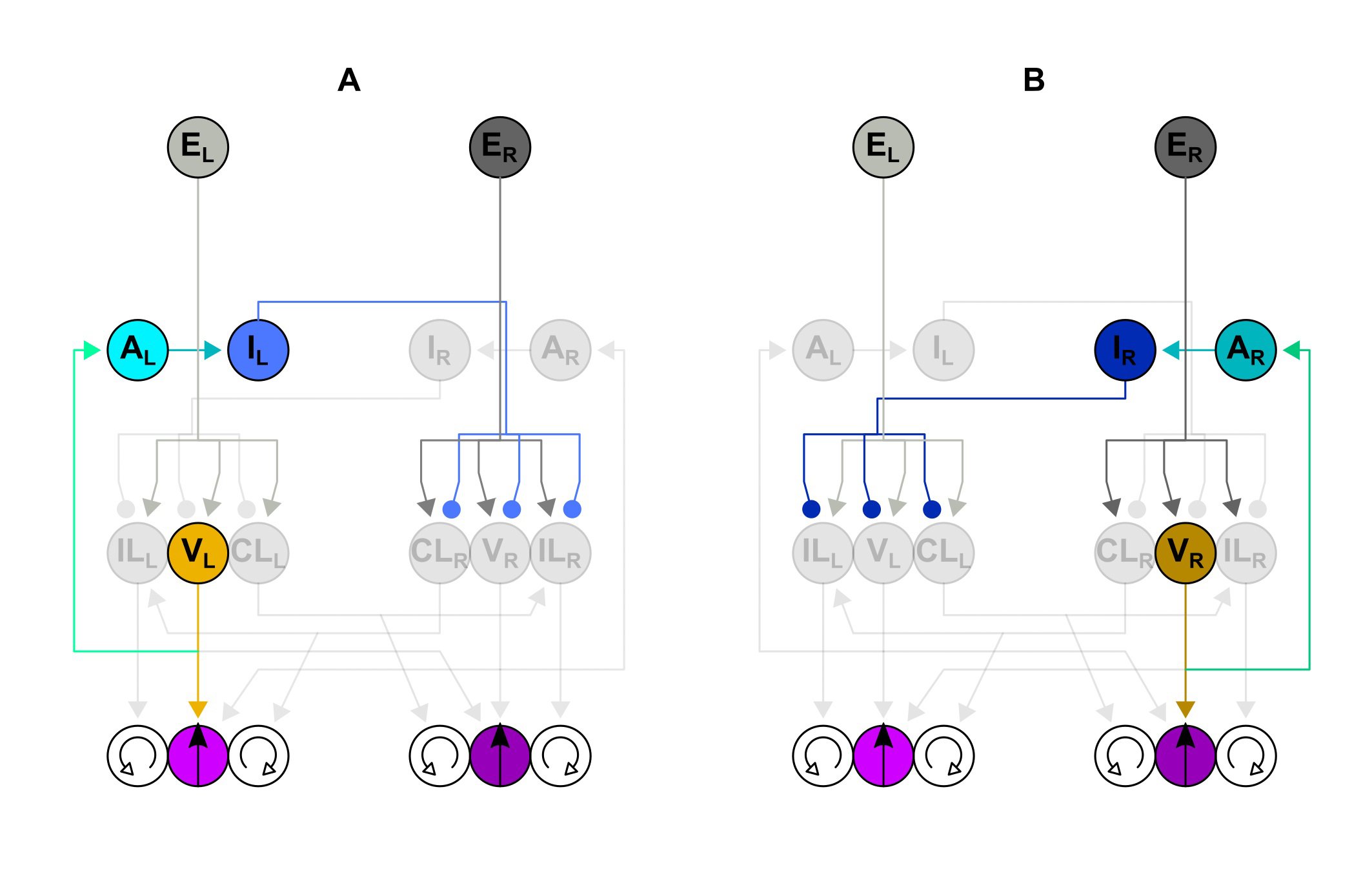

Supplement: Supplementary Figure 1 — Comprehensive network: Central Pattern Generator pathway. (A) The left-hand pathway (the colorful neurons and connections). The velocity neuron V drives the adapting neuron A, which in turn drives the inhibitory neuron I. I's output then inhibits the contra-lateral descending neurons. (B) The right-hand pathway. [file Image_1.jpg]

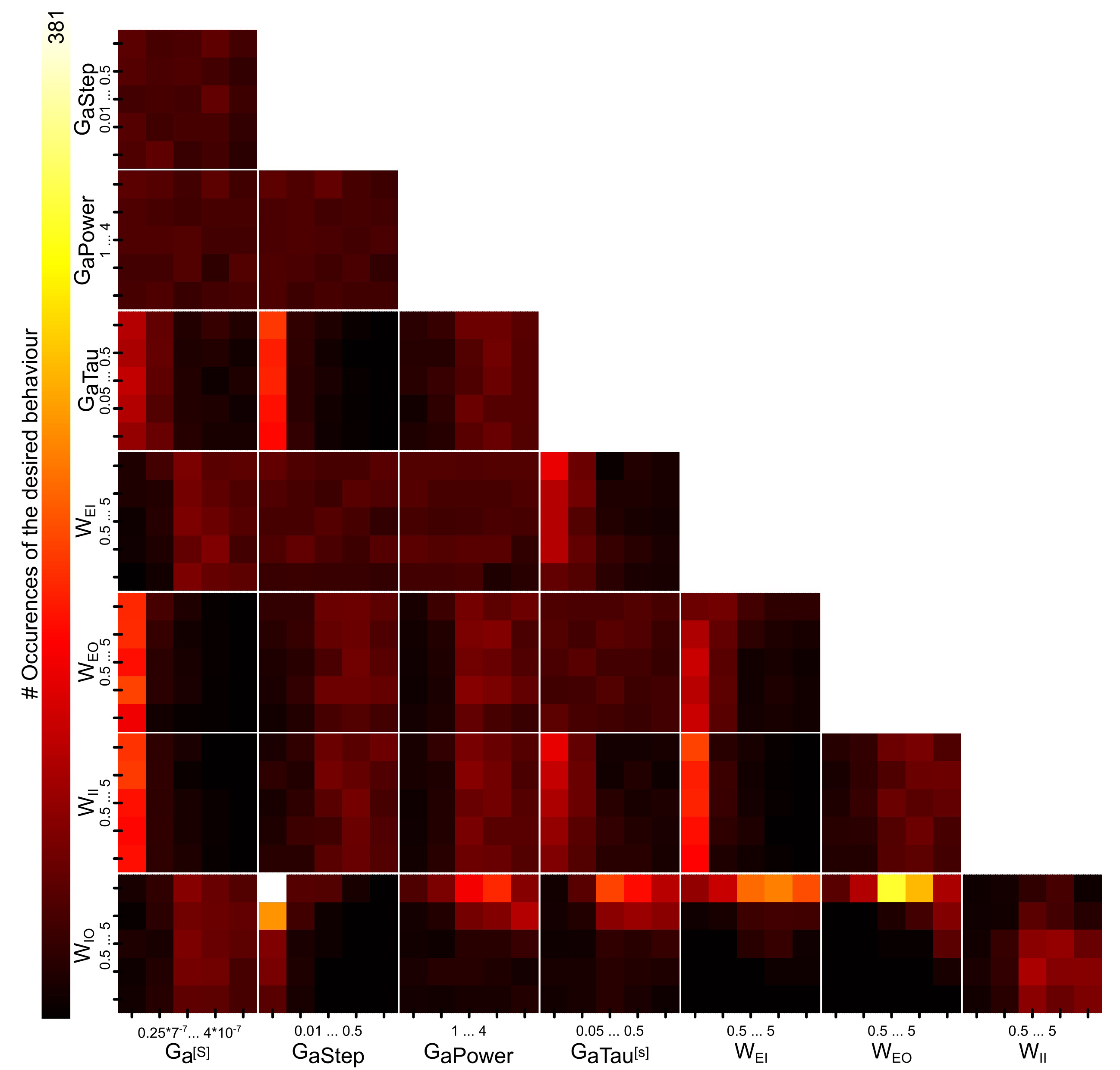

Supplement: Supplementary Figure 2 — Parameter combination relationships producing the desired behaviors in the Core network. Desired behavior: With low symmetric input the trajectory is strongly zig-zagging, with high input barely zig-zagging (see Figure 4Ba). The heatmaps show the network parameters plotted against each other in pairs. Some of the parameter pairs show clusters of combinations that are more likely to lead to the desired behavior. Many pairs show no clustering. [file Image_2.jpg]
